# Exogenous Lipoid Pneumonia Caused by E‐Cigarette Use Following Exposure to Oil Spray and Waterproofing Spray: A Case Report

**DOI:** 10.1002/ccr3.72520

**Published:** 2026-04-14

**Authors:** Ikuo Wakamatsu, Masakiyo Yatomi, Yuya Kuroiwa, Masataka Toyoda, Hiiru Sawada, Shun Inoue, Daisuke Shimizu, Shogo Uno, Chiharu Hanazato, Tomomi Masuda, Koichi Yamaguchi, Haruka Aoki‐Saito, Yosuke Miura, Hiroaki Tsurumaki, Yasuhiko Koga, Noriaki Sunaga, Masanao Saio, Takeshi Hisada, Isao Ito

**Affiliations:** ^1^ Division of Allergy and Respiratory Medicine, Integrative Center of Internal Medicine Gunma University Hospital Maebashi Gunma Japan; ^2^ Laboratory of Histopathology and Cytopathology, Department of Laboratory Sciences Gunma University Graduate School of Health Sciences Maebashi Gunma Japan; ^3^ Department of Rehabilitation Sciences Gunma University Graduate School of Health Sciences Maebashi Gunma Japan

**Keywords:** e‐cigarette, exogenous lipoid pneumonia, oil spray, waterproofing spray

## Abstract

Organic solvents contained in oil and waterproofing sprays, along with glycerin and propylene glycol found in e‐cigarettes, have been reported to trigger exogenous lipoid pneumonia. We report a 30‐year‐old male who developed lipoid pneumonia following exposure to paraffin‐based oil spray, waterproofing spray, and subsequent e‐cigarette use. Initial evaluation suggested occupational asthma due to elevated IgE and fractional exhaled nitric oxide levels, but inhaled corticosteroid therapy was ineffective. Chest computed tomography eventually revealed diffuse ground‐glass opacities, and bronchoalveolar lavage fluid analysis demonstrated lipid‐laden macrophages, confirming the diagnosis. Component analysis indicated that e‐cigarette vapor containing glycerin and propylene glycol was the primary trigger, whereas occupational exposures contributed minimally. The patient was managed with observation without steroid therapy, and symptoms resolved over 4 months with lifestyle modifications, including cessation of e‐cigarette use. This case highlights the combined impact of occupational and lifestyle factors in exogenous lipoid pneumonia and emphasizes the importance of assessing inhaled substances, workplace environment, and e‐cigarette use to determine the cause and guide exposure control.

## Key Clinical Message

A patient who smoked an electronic cigarette developed exogenous lipoid pneumonia following a lung injury caused by the inhalation of oil and waterproofing sprays at his workplace. Analyzing the components of inhaled substances and assessing the workplace environment may aid in clarifying the mechanism of lipoid pneumonia development.

## Graphical abstract

A man in his 30s working in furniture painting presented to our hospital with dyspnea. He was initially diagnosed with occupational asthma and treated accordingly, but his condition did not improve; ground‐glass opacities were observed on CT. He was admitted in December and underwent bronchoscopy, which revealed findings consistent with lipoid pneumonia. Prior to admission, the patient had stopped furniture painting and quit smoking conventional cigarettes, but had been using e‐cigarettes for three weeks. After two hospitalizations for observation, his dyspnea improved, the ground‐glass opacities on CT resolved, and the KL‐6 levels decreased.

The patient was diagnosed with exogenous lipoid pneumonia caused by exposure to e‐cigarette solvents following lung injury from oil‐based or waterproof sprays. The cause was identified by assessing the ingredients of all inhaled solvents.

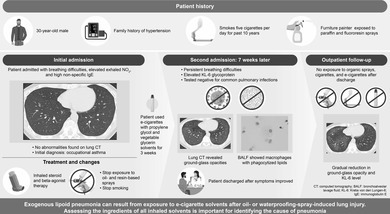

## 1 Introduction

Exogenous lipoid pneumonia is characterized by the presence of lipid‐laden macrophages in alveolar spaces [1]. This lung condition develops from the inhalation or aspiration of paraffin‐containing oil sprays and can be classified into acute and chronic types: acute exogenous lipoid pneumonia occurs when a large amount of lipid is accidentally aspirated over a short period, whereas chronic exogenous lipoid pneumonia results from prolonged, repetitive inhalation exposure to oil [1]. Computed tomography (CT) findings and the detection of lipid‐laden macrophages in sputum or bronchoalveolar lavage fluid (BALF) are important for diagnosing exogenous lipoid pneumonia [2, 3]. Recently, e‐cigarette use has been reported to cause lipoid pneumonia [4]. Furthermore, previous studies demonstrated that the inhalation of waterproofing sprays may cause acute lung injury (waterproof spray‐induced lung injury [WALI]) and that heating and smoking may exacerbate and prolong the condition [5, 6]. As described above, the inhalation of oil or waterproofing sprays and the use of e‐cigarettes can potentially cause lung injury and lipoid pneumonia. We encountered a case in which lipoid pneumonia developed following exposure to and inhalation of oil spray, waterproof spray, and e‐cigarette vapor. The patient was placed under observation without steroids and recovered 4 months later. We analyzed and investigated the components of inhaled substances that affected the onset of lipoid pneumonia.

## 2 Case History/Examination

**FIGURE 1:**
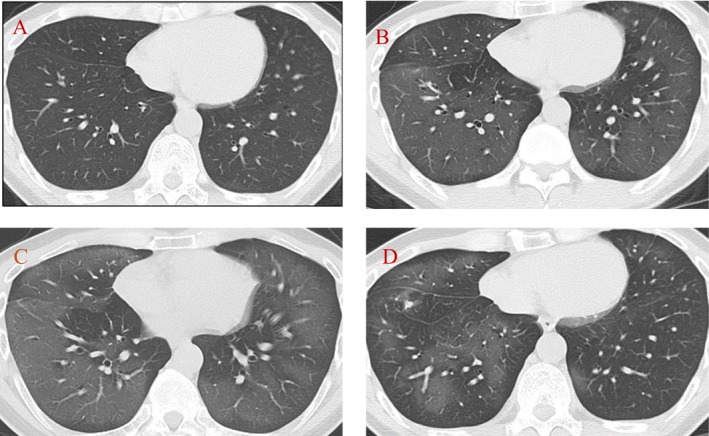
Progression of computed tomography findings. (A) During the first visit to our hospital, no abnormal findings were observed in either lung. (B) During the first hospitalization in December 2020, ground‐glass opacities appeared with areas of geographic sparing in both lungs. (C) During the second hospitalization in January 2021, ground‐glass opacities remained in both lungs. (D) In February 2021, ground‐glass opacities began resolving in both lungs.

The patient was a 38‐year‐old male with a history of hay fever and a family history of hypertension on his father's side. He had no significant medical history and took no regular medications. In early August 2020, the patient began painting furniture. Although the patient used a mask with a filter at work, he used organic solvents such as a paraffin‐containing oil spray and a fluorine resin‐containing coating agent (waterproofing spray) in a poorly ventilated environment. After painting, he dried the paint using a hair dryer. In early September 2020, the patient experienced breathing difficulties and visited a local clinic. Suspecting intractable respiratory disease, the clinic referred the patient to our hospital on October 12. At his first visit to our hospital, chest CT revealed no abnormal findings in the lungs (Figure 1A). The patient's fractional exhaled nitric oxide (FeNO) concentration and nonspecific immunoglobulin E (IgE) levels were high at 37 ppb and 1082 IU/mL, respectively. The attending physician suspected occupational asthma and initiated treatment with an inhaled corticosteroid/long‐acting beta‐agonist (ICS/LABA). The patient had been smoking approximately five cigarettes per day since the age of 20 years; nevertheless, the physician instructed smoking cessation and suggested a change in workplace. The patient immediately changed his work duties and stopped using oil and waterproofing sprays; however, he secretly smoked e‐cigarettes for 3 weeks as a substitute for traditional cigarettes. In November, he stopped smoking e‐cigarettes after experiencing worsened shortness of breath. During his revisit to the hospital on December 4, his shortness of breath persisted, and chest CT revealed diffuse ground‐glass opacities with areas of geographic sparing in both lungs (Figure 1B). The patient was admitted on December 9 for further investigation and treatment.

## 3 Differential Diagnosis, Investigations, and Treatment

At admission, the patient was conscious and alert, with a temperature of 36.5°C, pulse rate of 76 beats/min, blood pressure of 143/92 mmHg, and SpO_2_ of 97% on room air. Physical examination revealed no abnormalities in heart or lung sounds. Blood test results indicated a white blood cell (WBC) count of 8100/μL (neutrophils: 71.5%, eosinophils: 3.9%, basophils: 0.2%, lymphocytes: 20.1%, monocytes: 4.3%), lactate dehydrogenase level of 317 U/L, C‐reactive protein level of 0.62 mg/dL, sialylated carbohydrate antigen (KL‐6) level of 931 IU/mL, surfactant protein D level of 379 ng/mL, IgE level of 1082 IU/mL, B‐type natriuretic peptide level of 3.8 pg/mL, β‐D‐glucan level of < 3.0, and interleukin‐2 receptor level of 705 U/mL. The test results for myeloperoxidase‐antineutrophil cytoplasmic antibody, proteinase 3‐antineutrophil cytoplasmic antibody, *Mycobacterium avium* complex antibody, T‐SPOT.*TB* commercial assay, severe acute respiratory syndrome coronavirus 2, and *Trichosporon asahii* antibody were all negative. The test results for specific IgE to isocyanates used in furniture paint (methylene diisocyanate IgE, methylene diphenyl diisocyanate IgE, and hexamethylene diisocyanate IgE) were also negative.

**FIGURE 2:**
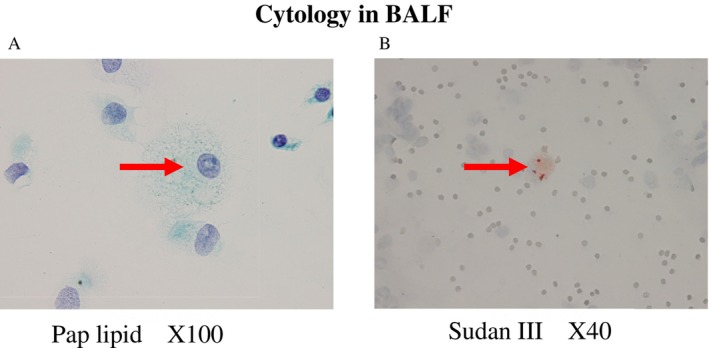
Cytology in bronchoalveolar lavage fluid. On Pap lipid staining (A) and Sudan III staining (B), foamy macrophages phagocytosed the lipid staining‐positive material.

Bronchoscopy was performed on day 13 of hospitalization. The BALF cell differential showed a high macrophage ratio, with a total cell count of 0.98 × 10^5^/mL (macrophages, 96.7%; lymphocytes, 0.7%; neutrophils, 0.7%; eosinophils, 1.9%; eosinophils, 0.0%). The CD4/CD8 ratio was 1.37. BALF cytology revealed scattered histiocytes that phagocytosed the lipid staining‐positive material (Figure 2A,B). The measured concentrations of benzene and toluene, which are the synthetic components of paraffin, in BALF were below the detection limits. Based on these results, the patient was diagnosed with exogenous lipoid pneumonia. Starting on Day 18 of hospitalization, a two‐night home exposure test was performed to differentiate it from hypersensitivity pneumonitis; no worsening of symptoms was observed.

As the patient's shortness of breath improved, he was discharged on Day 22. However, at a follow‐up visit on January 12, 2021, shortness of breath persisted, and high‐resolution CT (HRCT) revealed an exacerbation of ground‐glass opacities (Figure 1C); therefore, the patient was readmitted on January 12, 2021. After readmission, his shortness of breath gradually improved, and he was discharged on Day 12.

## 4 Outcome and Follow‐Up

**FIGURE 3:**
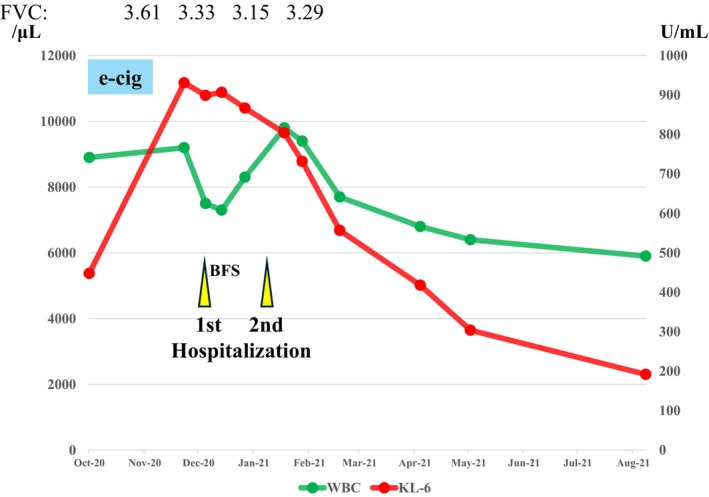
Clinical course and trends in laboratory values. After visiting our hospital in October 2020, the patient stopped smoking cigarettes and changed workplaces. However, electronic cigarettes were used for 3 weeks. KL‐6 levels showed an upward trend, whereas white blood cell (WBC) count did not. Bronchoscopy was performed during the first hospitalization in December 2020, and lipoid pneumonia was diagnosed. Following the second hospitalization in January 2021, a decreasing trend in both KL‐6 levels and WBC count and a reduction in dyspnea were observed. WBC, white blood cell; KL‐6, sialylated carbohydrate antigen; e‐cig, e‐cigarette; BFS, bronchofiberscopy; FVC, forced vital capacity.

During the observation period, the WBC count and KL‐6 levels were measured. The WBC remained at 8000/μL, whereas the KL‐6 levels increased; however, both decreased after February 2021. In addition, forced vital capacity (FVC) in pulmonary function tests showed a declining trend from November 2020 to January 2021, but an improvement was observed in February 2021 (Figure 3). Subsequently, his job duties were changed, and strict guidance on smoking cessation was provided. During outpatient follow‐up, the ground‐glass opacities on CT resolved, and the KL‐6 levels normalized (Figures 1D and 3). Steroids were not administered in the present case.

## 5 Discussion

This report describes a rare case in which a patient developed respiratory distress after using a paraffin‐based oil spray and a waterproof spray in a poorly ventilated environment, subsequently smoked e‐cigarettes, and was ultimately diagnosed with exogenous lipoid pneumonia. At the initial visit, IgE and FeNO levels were elevated, leading to the suspicion of occupational asthma and the initiation of treatment. However, treatment with ICSs was ineffective. Furthermore, results for specific IgE to isocyanates were negative, ruling out isocyanate‐induced hypersensitivity pneumonitis. Following e‐cigarette use, chest CT revealed bilateral ground‐glass opacities, and bronchoscopy showed the presence of lipid‐laden macrophages in BALF. The chronic type of exogenous lipoid pneumonia can sometimes show lipid‐laden macrophages. These findings, in conjunction with the clinical data, established a diagnosis of exogenous lipoid pneumonia.

Three inhaled substances were considered possible causative agents of both the patient's respiratory distress and exogenous lipoid pneumonia: oil spray, waterproof spray, and e‐cigarette vapor. Petroleum‐based solvents such as toluene, xylene, methyl ethyl ketone, and benzene, which are found in oil sprays, can reportedly cause exogenous lipid pneumonia [7]. In the present case, analysis of the components of the oil spray used in the patient's workplace revealed the presence of toluene, xylene, methyl ethyl ketone, and benzene (Table 1). The patient had avoided exposure to oil spray since October, and the toluene and benzene concentrations in BALF had been below the detection limit. Therefore, the influence of oil spray on exogenous lipoid pneumonia in this case was determined to be low.

**TABLE 1:** Components of oil‐based two paint sprays.

Product A		Product B	
Component	Proportion	Component	Proportion
Toluene (%)	36.83	xylene isomer mixture (%)	2.19
Isopropyl alcohol (%)	5–10	ethyl acetate (%)	20–25
Ethyl acetate (%)	10–15	butyl acetate (%)	30–35
Methyl ethyl ketone (%)	20–25	propylene glycol (%)	1–5
Propylene glycol, monomethyl ether (%)	5–10	methyl ether acetate, ethylbenzene (%)	2.19

*Note:* In Product A and Product B, petroleum‐based solvents such as toluene, xylene, methyl ethyl ketone, and benzene can cause exogenous lipoid pneumonia.

Fluororesin, a component of waterproof sprays, is associated with lung injury [8, 9]. Pathologically, fluororesin is thought to antagonize surfactant activity within the alveoli owing to its water‐repellent properties, causing diffuse alveolar collapse and possibly progressing to pneumonia [10]. Typical waterproof sprays consist of three components: a water‐repellent agent (fluororesin or silicone resin), a solvent (n‐heptane or ethyl acetate), and a propellant (propane or butane). The sprays used by the patient in the present case contained n‐heptane (97%), acetone (< 2%), and fluororesin (< 2%).

WALI generally presents with dyspnea, cough, and fever, and its HRCT findings resemble those of pulmonary congestion or *Pneumocystis* pneumonia [11]. Although our patient may have initially developed WALI, chest CT at the first visit revealed no abnormal findings in either lung (Figure 1A). A previous study comparing early‐ and late‐improving cases of WALI reported that late‐improving cases exhibited diffuse lung damage throughout all lung fields, significantly decreased serum WBC counts, and elevated KL‐6 levels [11]. In our case, the mildly decreased WBC count and mildly elevated KL‐6 levels at the initial visit suggested a tendency toward delayed improvement of WALI (Figure 3).

Wheezing was not detected on auscultation; nonetheless, the presence of an allergic predisposition on blood testing led to the suspicion of occupational asthma. The patient was treated with ICS/LABA combination therapy; however, at the follow‐up visit on December 4, 2021, dyspnea did not improve. Upon further inquiry, the patient reported using e‐cigarettes for 3 weeks after the initial visit on October 12. After e‐cigarette use, CT revealed new ground‐glass opacities in both lungs (Figure 1B), along with increased KL‐6 levels and decreased FVC (Figure 2). These findings strongly suggested that the 3‐week e‐cigarette use was the main trigger for the onset of exogenous lipoid pneumonia.

Components of e‐cigarettes have been implicated in the development of exogenous lipoid pneumonia [12]. E‐cigarette or vaping use‐associated lung injury (EVALI), which has been reported in the United States [13], may share a mechanism similar to that of oil spray‐induced exogenous lipoid pneumonia [14]. Vitamin E acetate is a major contributor to EVALI [15]. Furthermore, glycerol and propylene glycol, which are essential for vapor production in e‐cigarettes, can cause airway inflammation and mucosal irritation [16]. Although the e‐cigarettes used by our patient did not contain vitamin E acetate, they contained plant‐derived glycerin and propylene glycol (Table 2), which were considered the primary causes of lipoid pneumonia in the patient.

**TABLE 2:** Ingredients of the two e‐cigarettes used by the patient.

	Product C	Product D
Propylene glycol (%)	30	40
Vegetable glycerin (%)	70	60

*Note:* Both Product C and Product D contained propylene glycol and glycerin, which can cause lipoid pneumonia.

Fortunately, the patient's condition did not deteriorate to a level that required oxygen therapy. Therefore, observation without steroid administration was chosen, and spontaneous resolution of exogenous lipoid pneumonia was achieved.

This case report highlights a rare instance in which exposure to oil‐based paint and waterproof spray resulted in respiratory dysfunction, followed by the onset of exogenous lipoid pneumonia triggered by e‐cigarette use. Exogenous lipoid pneumonia can arise from a combination of occupational and lifestyle‐related factors, including exposure to workplace chemicals and e‐cigarette use, emphasizing the importance of thorough post‐WALI lifestyle guidance and control.

## 6 Conclusion

Pulmonary injury can occur following the inhalation of oil and waterproof sprays, and subsequent e‐cigarette use may lead to the development of exogenous lipoid pneumonia. The onset of exogenous lipoid pneumonia may involve multiple inhaled components. Analyzing the ingredients of the inhaled substances and assessing the workplace environment are important for identifying the cause.

## Author Contributions

**Ikuo Wakamatsu:** conceptualization, investigation, writing – original draft. **Masakiyo Yatomi:** investigation, visualization, writing – original draft, writing – review and editing. **Yuya Kuroiwa:** investigation, resources. **Masataka Toyoda:** investigation, resources. **Hiiru Sawada:** investigation, resources. **Shun Inoue:** investigation, resources. **Daisuke Shimizu:** investigation, resources. **Shogo Uno:** resources. **Chiharu Hanazato:** resources. **Tomomi Masuda:** resources. **Koichi Yamaguchi:** validation. **Haruka Aoki‐Saito:** validation. **Yosuke Miura:** validation. **Hiroaki Tsurumaki:** validation. **Yasuhiko Koga:** methodology, validation. **Noriaki Sunaga:** methodology, validation. **Masanao Saio:** resources, visualization. **Takeshi Hisada:** writing – review and editing. **Isao Ito:** supervision, writing – review and editing.

## Funding

The authors have nothing to report.

## Ethics Statement

The authors have nothing to report.

## Consent

The patient provided detailed written informed consent for the incorporation of his medical history, images, laboratory reports, and personal information into the case report.

## Conflicts of Interest

The authors declare no conflicts of interest.

## Data Availability

The data supporting the findings of this study are available from the corresponding author upon reasonable request.
